# Retrospective evaluation of the utility of two-step surgery for facial basal cell carcinoma and squamous cell carcinoma

**DOI:** 10.3389/fsurg.2022.915731

**Published:** 2022-09-07

**Authors:** Shiro Iino, Natsuki Baba, Takumi Hasegawa, Hiroshi Kasamatsu, Noritaka Oyama, Takahiro Tokunaga, Minoru Hasegawa

**Affiliations:** ^1^ Department of Dermatology, Division of Medicine, Faculty of Medical Sciences, University of Fukui, Fukui, Japan; ^2^ Medical Research Support Center, University of Fukui Hospital, Fukui, Japan; ^3^ Research Promotion Office, Shinseikai Toyama Hospital, Toyama, Japan

**Keywords:** facial skin cancers, basal cell carcinoma, squamous cell carcinoma, two-step surgery, dermal regeneration template

## Abstract

In older patients with facial basal cell carcinoma (BCC) or squamous cell carcinoma (SCC), surgery should be aimed to reduce treatment-related sequelae and burden with achieving local tumor care. Therefore, we adopted a two-step surgery (TSS) involving the application of a dermal regeneration template onto the skin defect after tumor resection and subsequent reconstruction by full-thickness skin grafting. We performed a detailed comparison of conventional one-step surgery (OSS) and TSS, including evaluation of local tumor curability, postoperative cosmetic and/or functional impairments, and patient burden. Forty-six patients who underwent TSS and 104 patients treated with OSS were retrospectively investigated. The cohort consisted of 77 men and 73 women (median age, 83 years). The BCC: SCC ratio was 56.7%: 43.3%. The tumor size and excision margin were significantly larger in the TSS group than in the OSS group (*p *= 0.03). The histopathological margin was positive after the first surgery in six cases, but was negative after additional resection in all cases, regardless of OSS or TSS. Local recurrence was not observed in this study. The frequency of postoperative sequelae (POS) in TSS was slightly lower than in OSS (17.4% vs. 27.9%, *p *= 0.16). A shorter average operation time per session was significantly associated with the location of the vertical defect [below adipose tissue vs. within adipose tissue, estimate: −0.28 (hour), *p *< 0.001] and surgical procedure [OSS vs. TSS, estimate: −0.13 (hour), *p *= 0.03] by multiple regression models. The ratio of general anesthesia was relatively lower in TSS than in OSS (9.8% vs. 17.3%, *p *= 0.12). Thus, TSS showed a good local curability and POS statistically equivalent to OSS, reducing the surgical burden, particularly shortening each operation time without any adverse events, despite the TSS group having significantly larger tumors than the OSS group. Since TSS is a simple procedure, it can be an outstanding option for facial BCC and SCC.

## Introduction

Non-melanoma skin cancers (NMSCs) are the most common among all types of malignancies. The American Cancer Society estimates suggest that nearly 200,000 people developed NMSCs, mostly basal cell carcinomas (BCCs) and squamous cell carcinomas (SCCs), in 2020 (1). NMSCs are generally curable, but their increasing incidence poses major medical healthcare and economic issues. Complete surgical removal of the tumor is the mainstay of the primary treatment for these cancers. However, the craniofacial area consists of several anatomical subunits with distinct properties (i.e., sensory and respiratory subunits), all of which are functionally and/or cosmetically indispensable. Therefore, complete surgical removal of the craniofacial skin tumors requires greater emphasis on functional preservation and cosmetic reconstruction than in other parts. Another major concern is that NMSCs mostly affect older individuals (2), necessitating minimization of the surgical burden and a focus on avoiding complications.

Micrographic surgeries (MSs), containing Mohs micrographic surgery (MMS) and slow-MMS, demonstrate the highest cure rates for several cutaneous malignancies. MMS is widely performed in Europe and the United States (3–5), allowing histological confirmation of tumor free-surgical margin intraoperatively and minimizing normal tissue defects (6). Slow-MMS, a modified method of MMS (7, 8), enables more accurate histological evaluation using formalin-fixed samples, though reconstruction is time-consuming. In Japan, however, surgeons performing MSs remain a minority, because of technical and scheduling issues for dermatologists and dermatopathologists. Thus, one-step surgery (OSS) has been widely accepted in Japan. The OSS procedure comprises three major steps; (i) the tumor resection with wide local excision (WLE), (ii) any immediate reconstruction (skin flap, skin grafting, open treatment, etc.), and (iii) later histological evaluation using serial transverse cross-sectioning (bread-loafing) or an equivalent method. However, the OSS has limitations to identify the exact site(s) of the residual tumor and minimize the sacrifice of surrounding normal tissue, when additional excision was needed. Moreover, time-consuming surgery often provides a physical and mental burden for elderly patients.

As an alternative approach other than MSs and OSS, two-step surgery (TSS) was conducted using 256 postoperative patients with BCC (9). It consisted of WLE covered with a dermal regeneration template in the first surgery, followed by any reconstruction methods in the second surgery. Thus, the original TSS represents an equal concept with slow-MMS, both requiring a waiting time until reconstruction and histological evaluation using formalin-fixed permanent specimens. However, little is known about the potential utility of TSS for facial skin tumors. We have previously reported the clinical benefit of TSS by combining the use of a dermal regeneration template, for a postoperative finger defect and subsequent reconstruction with full-thickness skin grafting (FTSG) in nail unit melanoma (10). The distinct two surgical steps in each patient were completed by local anesthesia alone and provided satisfactory cosmetic outcomes without local recurrence and adverse events. In this report, we extended the TSS procedure to the treatment of facial BCC and SCC, to retrospectively assess the clinical advantages of TSS.

Our findings showed that this surgical method not only ensures reliable resection of the tumor, but also tends to reduce the surgical burden, yielding postoperative sequelae (POS) equivalent to OSS.

## Materials and methods

### Patients

We primarily enrolled 159 patients with BCC or SCC of the face who had undergone operations at the Department of Dermatology, University of Fukui Hospital from 2012 to 2019. In all cases, conclusive diagnoses were obtained by histopathological analyses of permanent specimens after resection. The cohort consisted of patients aged at least more than 20 years. Of these, we excluded two patients with genodermatoses associated with high incidence of skin malignancy (xeroderma pigmentosa), and seven cases who underwent reconstruction procedures other than FTSG in TSS (see the following section). Thus, 150 patients with facial BCC (*n* = 85) or SCC (*n* = 65) were retrospectively investigated (Table 1).

**Table 1:** Characteristics of the 150 patients treated with OSS or TSS.

	Surgical procedure			
Variable	OSS	TSS	Total	*p*
Sex				0.55^b^
Male	51 (49.0)^a^	25 (54.3)	76 (50.7)	
Female	53 (51.0)	21 (45.7)	74 (49.3)	
Age				0.63^b^
Median (IR), years	83 (43–97)	83 (57–98)	83 (43–98)	
Tumor type				0.98^b^
BCC	59 (56.7)	26 (56.5)	85 (56.7)	
SCC	45 (43.3)	20 (43.5)	65 (43.3)	
Tumor diameter				**0.03** ^c^
Median (IR), mm	11 (7–18)	16 (12.8–24.3)	13 (8–20)	
Excisional margin				**0.03** ^c^
Median (IR), mm	2 (2–3.75)	4 (3–6)	3 (2–5)	
Vertical defect location				0.32^b^
Within adipose	81 (77.9)	39 (84.8)	120 (80.0)	
Below adipose	23 (22.1)	7 (15.2)	30 (20.0)	
Anatomical site				0.15^b^
Mask area	67 (64.4)	35 (76.1)	104 (69.3)	
Others	37 (35.6)	11 (23.9)	46 (30.7)	

OSS, one-step surgery; TSS, two-step surgery; IR, interquartile range; BCC, basal cell carcinoma; SCC, squamous cell carcinoma. *p *< 0.05.

^a^ *n* (%).

^b^ *χ*^2^ test.

^c^ Student’s *t*-test.

Value with *p* < 0.05 were bold.

This study was approved with being made its outline publicly available in advance, by the Ethics Committee of the University of Fukui Hospital (approval number: 20200181).

### The two-step surgery procedure

The TSS in this study referred to the reconstruction method limited to FTSG using the dermal regeneration template Integra® (Integra Life Sciences Co., NJ, USA), and was applied to the following patients: (i) tumor boundaries are clinically unclear, and (ii) tumors that take a long operation time depending on the size or location, and supposed to require general anesthesia, being a high risk for elderly or patients with underlying diseases. The primary excision margin was set to obtain the complete negative margin of the tumor, regardless of OSS or TSS.

**Figure 1:**
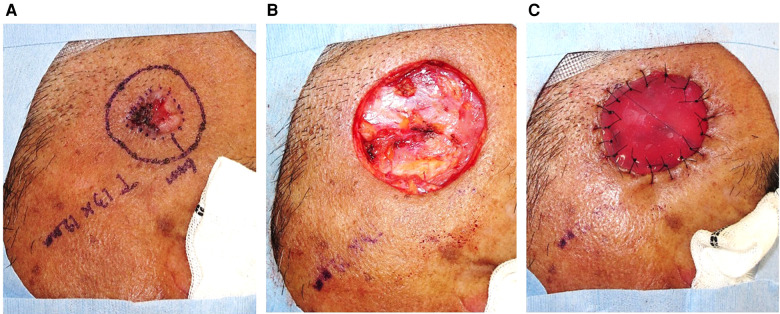
Facial SCC was resected with a standard excision margin, followed by covering with Integra®.

**Figure 2:**
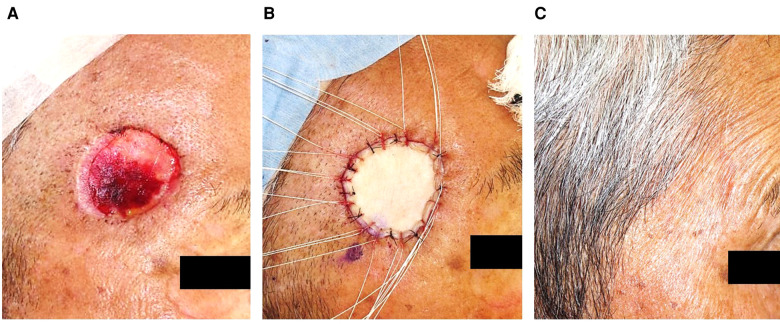
After the Integra® had been removed, the defect was reconstructed by FTSG, without noticeable sequelae a year after the second surgery.

The detailed procedures of TSS for SCC were as follows (11). In the first step, facial SCC (Figure 1A) was resected with a standard excision margin based on the recommended criteria in Japan (12) (Figure 1B), followed by covering with Integra® (Figure 1C). The resected sample was marked by a one-point suture, and the surgical part was photographed before and immediately after excision. These steps clarified the positional relationship between the tumor and the post-surgical skin defect during additional resections in cases wherein a positive histological margin was confirmed histologically. The sample was formalin-fixed for hematoxylin-eosin staining to microscopically check the tumor-free margin, in a preparation step called “bread-loafing” similar to that used for OSS. After confirming complete tumor resection histologically, the Integra® sheet was removed from the wound bed where granulation was sufficient without contraction (Figure 2A), and the postoperative skin defect was finally reconstructed by FTSG as the second step (Figure 2B). If the surgical margin was positive histopathologically, additional excision was performed at the time of the second surgery, which finally ensured complete resection. In our cohort, no local recurrence, metastasis, or POS could be confirmed at least 1 year after the second operation (Figure 2C). In addition, each of the two operation steps was completed within an hour under local anesthesia. During the period between the first and second operations in TSS, the patients experienced no persistent pain or bleeding, except just after the operations.

### Endpoints and outcomes

We compared TSS with conventional OSS to examine the effectiveness of TSS in terms of the following endpoints: (i) local tumor curability, (ii) postoperative cosmetic and/or functional impairment, and (iii) patient burden.

We initially evaluated the histopathological margin (HM) after tumor resection (primary or additional) and local tumor recurrence (LTR) as outcomes to assess local tumor curability. Regional lymph node metastasis and in-transit metastasis were not included in LTR, but lesions that were indistinguishable from skin metastases due to the close proximity to the primary lesion were considered to indicate LTR.

To assess postoperative functional or cosmetic impairment, POS was evaluated on the basis of the medical records at the patient’s last visit. If the records contained any description of sequelae (irreversible or reversible) recognized by a doctor and/or patient’s complaint, the case was considered positive for POS. The relevant terms in the medical records included words such as “unmatched color,” “unmatched texture,” “deformation of the skin,” “scar contracture,” “ulceration,” and “restriction of local movement.” As a secondary analysis, OSS was classified by the reconstruction method, and the outcomes for each method were compared to those of TSS.

Finally, the operation time and general anesthesia rate (GAR) for each surgical procedure were identified as outcomes to assess patient burden. For cases involving TSS, the average time of the two surgeries was obtained and compared to the time required in cases involving OSS. GAR adopted the ratio of general anesthesia to the total number of operations (two operations per case for TSS).

### Statistical analysis

In the present study, we investigated the distribution of the following clinical variables: sex, age, tumor type, tumor diameter, excision margin, vertical defect, and anatomical site. The *χ*^2^ test and Student’s *t*-test were performed for comparisons between surgical procedures (OSS or TSS) and variables or outcomes (HM, POS, operation time, and GAR). The POS associated with each reconstruction method used in OSS were compared with those associated with TSS by the *χ*^2^ test. In addition, multiple regression models were prepared to determine the extent to which each variable was related to operation time. We followed standard methods to estimate the number of independent variables for multiple regression, with at least 15 samples needed for each included independent variable. Since there were 150 cases, we included a maximum of 10 variables in our analysis. The variance inflation factor was used to check for multicollinearity. Among the variables listed above, sex was excluded since it was considered to not affect the operation time clinically, and the analysis was performed using all the other variables. Regarding GAR for TSS, the two operations were counted as separate procedures, and the total ratio of them was compared with the values for OSS by the *χ*^2^ test. Statistical analysis was performed using JMP software® (ver. 14. 2. 0), and *p *< 0.05 was considered statistically significant.

## Results

### Patient characteristics

This retrospective study included 150 patients with facial BCC and SCC, of which 104 underwent OSS (69.3%) and 46 underwent TSS (30.7%). Table 1 shows the characteristics of each group. The cohort consisted of 76 (50.7%) male and 74 (49.3%) female patients with a median age of 83 years (range, from 43 to 98 years). The ratio of BCC to SCC was 85 (56.7%):65 (43.3%). The median tumor diameter was 13 mm (range, 8–12 mm), and the median excision margin of the tumor was 3 mm (range, 2–5 mm). The vertical skin defect after tumor resection remained within the adipose tissue in 120 (80.0%) patients, but the defect was deeper than adipose tissues (reached muscles, cartilage, and mucosa) in 30 (20.0%) patients. Anatomical sites included the mask area (central face, eyelid, eyebrows, periorbital, nose, lips, chin, mandible, preauricular skin, temple, and ears) in 104 (69.3%) cases and other areas (cheeks and forehead) in the remaining 46 (30.7%) cases. The tumor diameter and excision margin were significantly longer in the TSS group than in the OSS group (*p *= 0.03). The proportion of cases with tumors in the mask area that were cosmetically or functionally difficult to resect and reconstruct was slightly higher in TSS than in OSS, but the difference was not statistically significant (*p *= 0.15). No other differences were observed in relation to the surgical procedure among the variables in the univariate analysis.

### Local tumor curability

We identified HM and LTR as the outcomes for assessing local tumor curability. No LTR-positive case was observed in our study. Table 2 presents the data for six cases that showed a positive HM at the first tumor resection, irrespective of the surgical procedures. Three patients (patients A, B, and C) were treated with OSS, of which two (patients A and C) did not receive additional excision due to the focal *in situ* lesions. Patient A did not show recurrence but died of tumor-unrelated disease at 31 weeks after surgery, while patient C has not shown relapse more than 4 years after the operation. Patient B received additional resection that yielded a negative HM, although he developed multiple cervical lymph node metastases 16 weeks post-surgery and eventually died of aspiration pneumonia 44 weeks after the second surgery. The remaining three patients who were treated with TSS (patients D, E, and F) underwent additional resection in the second surgery, which yielded negative HM. Although these results suggest excellent local tumor curability in the whole study, there was no clear significant difference between OSS and TSS.

**Table 2:** The six cases showing a positive histopathological margin after the first surgery.

Patient (age in years), sex	Tumor type (size), mm	Site	SP	AE	HM after AE
A (87), male	SCC (18)	Nose	OSS	No	–
B (93), male	SCC (40)	Cheek	OSS	Yes	Negative
C (89), female	SCC (10)	Eyelid	OSS	No	–
D (82), female	BCC (4)	Nose	TSS	Yes	Negative
E (74), male	SCC (24)	Cheek	TSS	Yes	Negative
F (86), male	SCC (11)	Eyelid	TSS	Yes	Negative

SP, surgical procedure; AE, additional excision; HM, histopathological margin; BCC, basal cell carcinoma; SCC, squamous cell carcinoma; OSS, one-step surgery; TSS, two-step surgery.

### Postoperative cosmetic and/or functional impairments

We conducted the *χ*^2^ test to determine whether surgical procedures affect POS as an outcome for postoperative functional or cosmetic impairments. Although TSS was associated with a lower incidence of POS than OSS, the difference was not significant (17.4% vs. 27.9%, *p *= 0.16) (Table 3). Other comparisons of POS in TSS versus OSS classified by reconstruction methods showed a significant difference only between TSS and OSS with open treatment (17.4% vs. 66.7%; odds ratio, 9.09; *p *< 0.01) (Table 3).

**Table 3:** Chi-square test for comparison of POS in TSS and OSS with various reconstruction procedures.

Surgical procedure	POS		OR (95% CI)	*p*
	Positive	Negative		
TSS	8 (17.4)^a^	38 (82.6)	1 (reference)	–
OSS	29 (27.9)	75 (72.1)	1.85 (0.76–4.35)	0.16
Flaps	8 (24.2)	25 (75.8)	1.52 (0.51–4.55)	0.14
FTSG	8 (33.3)	16 (66.7)	2.38 (0.76–7.69)	0.46
STSG	0 (0.0)	1 (100)	–	0.54
Simple stitching	1 (3.6)	27 (96.4)	0.18 (0.02–1.49)	0.06
Open treatment	12 (66.7)	6 (33.3)	9.09 (2.78–33.33)	**<0.01**

POS, postoperative sequelae; OR, odds ratio; CI, confidence interval; TSS, two-step surgery; OSS, one-step surgery; FTSG, full-thickness skin grafting; STSG, split-thickness skin grafting. *p *< 0.05.

^a^ *n* (%).

Value with *p* < 0.05 were bold.

### Patient burden

Operation time and rates of single general anesthesia were used as one of the indicators for patient surgical burden. Then, we used a multiple regression analysis to determine whether the surgical method and the variables listed in Table 1, excluding sex, affected the operation time. In the preliminary Student’s *t*-test, a significant difference was observed in the operation time between surgical procedures [OSS vs. TSS, mean difference −0.26 (hour), *p *= 0.03] (Table 4). In multiple regression models, surgical procedure [OSS vs. TSS, estimate: −0.13 (hour), *p *= 0.03] and location of the vertical defect [below adipose tissue vs. within adipose tissue, estimate: −0.28 (hour), *p *< 0.001] were the variables that were significantly related to operation time (Table 5). Surgery under general anesthesia was performed in 18 of the 104 OSS procedures (17.3%) and nine of the 92 procedures performed under TSS (9.8%, Table 6). GAR tended to be lower in TSS cases than in OSS cases, but the difference between the two groups was not statistically significant (*p *= 0.12) (Table 6). The median interval between the first and second operations in TSS was 33 days (interquartile range, 28–37 days), and there were no cases with adverse events that required any treatment, such as infection, bleeding, or hematoma, in this period.

**Table 4:** Student’s *t*-test for comparison of operation time per session between surgical procedures.

Operation time, hour	Surgical procedure		*p*
	OSS	TSS	
Median	1.20	1.01	
IR	0.71–1.68	0.87–1.18	
MD (95% CI)	0 (reference)	−0.26 (−0.48 to −0.03)	**0.03**

OSS, one-step surgery; TSS, two-step surgery; IR, interquartile range; MD, mean difference; CI, confidence interval. *p *< 0.05.

**Table 5:** Multiple regression models for operation time per session and variables including surgical procedure.

Variable	Estimate	95% CI	*p*
Intercept	1.39	0.60–2.19	<0.01
Surgical procedure			
OSS	0 (reference)		
TSS	−0.13	−0.24 to −0.01	**0.03**
Age			
Per year	−0.002	−0.012–0.007	0.63
Tumor type			
SCC	0 (reference)		
BCC	0.023	−0.11–0.007	0.73
Tumor diameter			
Per mm	0.003	−0.008–0.013	0.61
Excisional margin			
Per mm	0.02	−0.04–0.09	0.48
Vertical defect location			
Below adipose tissue	0 (reference)		
Within adipose tissue	−0.27	−0.41 to −0.14	**<0.001**
Anatomical site			
Others	0 (reference)		
Mask area	0.0002	−0.12–0.12	0.10

CI, confidence interval; TSS, two-step surgery; OSS, one-step surgery; BCC, basal cell carcinoma; SCC, squamous cell carcinoma. *p *< 0.05.

Value with *p* < 0.05 were bold.

**Table 6:** Rates of local and general anesthesia for each surgical procedure in the *χ*^2^ test.

	Surgical procedure		*p*
	OSS	TSS	
Anesthesia			
Local	86 (82.7)^a^	83 (90.2)	
General	18 (17.3)	9 (9.8)	0.12
OR (95% CI)	1 (reference)	0.52 (0.22–1.22)	

OSS, one-step surgery; TSS, two-step surgery; OR, odds ratio; CI, confidence interval. *p *< 0.05.

^a^ *n* (%).

## Discussion

Our retrospective study demonstrated that TSS is comparable to OSS in safety and usefulness for the treatment of BCC and SCC on the face. No local recurrence was observed throughout the study, and all TSS cases who had a positive HM after the first resection showed a negative HM after the second surgery. In addition, the frequency of postoperative cosmetic and/or functional problems in the TSS group was almost similar to that in the OSS group. The individual operation time in the TSS group was significantly shorter than that of OSS, and there were no cases with any adverse events during the period between the two surgeries.

Surgery for malignant cutaneous tumors can be broadly divided into methods involving MSs and WLE, which is the procedure that removes tumors with a certain margin. MSs contain normal MMS and slow-MMS, and WLE contains OSS and TSS. The relationship between the two procedures in MSs, MMS and slow-MMS, can be recognized as those between OSS and TSS in WLE, respectively. That is, slow-MMS and TSS have in common that tumor tissue is evaluated with permanent specimens before reconstruction. In the past, there have been reports comparing WLE and MMS (13) or WLE and slow-MMS (14, 15) in the treatment of various cutaneous malignancies, but there is only one report comparing OSS and TSS by Goto et al. (9) The report described a large number of Japanese cases with BCC (*n* = 256) throughout the body who were treated with TSS and any reconstruction methods. In this study, the TSS group showed no recurrence, although the local recurrence rate did not differ significantly between OSS and TSS. Therefore, they concluded that TSS may be useful for BCCs with sites and histological types prone to recurrence. Our study differs from their report in terms of focusing on facial BCC and SCC. All our TSS cases were temporally stitched with a dermal regeneration template, Integra®, which is less likely to cause contracture due to extracellular matrix additives (e.g., glycosaminoglycans, etc.), followed by reconstruction using only FTSG. In addition, we performed a detailed comparison of OSS and our TSS in order to evaluate positive HMs, LTR, POS, and the physical burden of surgery using operation time and GAR.

Surgical treatment of facial BCC and SCC needs to achieve curative resection of the primary tumor. The NCCN guidelines® have proposed the concept of “Peripheral and deep en face margin assessment (PDEMA)” to evaluate excision margins for BCC and SCC (16, 17) and recommended the following criteria: (1) the entire margin surface of the surgical specimen is microscopically visualized and histopathologically analyzed for the presence of cancer; the margin surface includes complete deep and peripheral margin; (2) the surgical specimen is oriented with respect to the surgical site and marked in a manner such that any positive margin identified in histopathologic analysis can be accurately located and re-excised; (3) the surgical margin of any re-excised tissue is again entirely visualized and oriented as above; this process is repeated till no further cancer is identified at the surgical margin or until further excision is not anatomically possible or not in the best interest of the patient; (4) the interval between the steps of this process is rapid enough to prevent significant size or shape changes in the wound bed (i.e., granulation, contraction) that would decrease the accuracy of orientation (17).

When we evaluated the TSS technique based on the PDEMA criteria, it did not meet criteria (1), (2), and (3), since TSS uses “bread-loafing” for cutting out specimens, similar to OSS. However, regardless of the surgical procedure, OSS or TSS, none of the cases showed LTR in our study. This may suggest that even if the specimen is prepared by “bread-loafing,” high local curability for the facial BCC and SCC can be obtained. In addition, despite positive HMs being seen in six cases (one OSS and three TSS), the additional resection provided negative HM and no recurrence in all cases during the follow-up, suggesting the equivalent local curability between OSS and TSS. Another advantage of TSS can maintain the relatively accurate orientation for re-excision as mentioned in PDEMA criteria (4). A dermal regeneration template Integra® used in TSS enriched glycosaminoglycans that act as extracellular matrices in the dermal interstitial and suppress the wound contraction (18, 19). On the other hand, OSS might cause a loss of orientation for re-excision depending on the reconstruction methods and offer further tissue loss by another wide excision and reconstruction. When required additional excision, therefore, TSS has an advantage, although OSS and TSS were both excellent results in HM and LTR.

Avoidance of cosmetic and functional impairments is an important consideration in facial skin tumor surgery. Our assessment using the final medical records showed that TSS was associated with a somewhat lower frequency of POS than OSS, although there was no statistically significant difference. However, the tumor size and excisional margin were significantly larger in TSS than in OSS. TSS comprises two simple surgical procedures without high technical skills rather than OSS with complex reconstruction such as skin flaps. It is therefore conceivable that shorter operation time and simplified procedure may affect the overall POS in patients who underwent surgery for facial SCC and BCC. Furthermore, our results demonstrated that the incidence of POS was significantly higher in patients with open treatment than in those with TSS. Certainly, open treatment is a more simple procedure than TSS and as excellent as TSS with additional excision for positive HM. TSS should be selected over open treatment for minimizing POS, especially in the treatment of anatomically delicate areas such as the face.

Since most BCCs and SCCs occur in older individuals, the surgical invasion should be minimized and less burdensome to them. To monitor these, we adopted operation time and anesthesia method as the outcomes to evaluate the patient burden during surgeries. The average time of two operations was used as the operation time in TSS. Multiple regression models identified the position of the vertical defect and surgical procedure as variables that significantly reduced operation time. In addition, general anesthesia tended to be used with slightly less frequency in TSS than in OSS, despite the significantly larger tumor and surgical margin. Thus, in some cases, a long-time surgery, like requiring general anesthesia, can be split into two short procedures with local anesthetics. However, even if the burden of every single surgery is smaller, the patient needs to wait with a postoperative ulcer on their face during the period between the two surgeries, which may carry the risk of infection. Although there were no cases of adverse events during the period between the two operations on TSS in our study, these things should be taken into consideration.

A major limitation of this study was a retrospective single-center study with a limited number of cases, in which the findings may have been influenced by various biases such as the patient’s background, tumor characteristics, or selection of the procedure. Indeed, the tumor diameter and excision margin were significantly larger in the TSS group than in the OSS group, which may be biased in choosing TSS. However, our study revealed no differences in HM positivity and LTR between OSS and TSS groups. This may allow consideration of smaller resection margins in TSS and needs to be estimated precisely in low-risk cases in the future.

Nonetheless, TSS can be an attractive option for the treatment of facial BCC and SCC. Depending on the patient’s background or characteristics of the tumor, TSS may be a good indication in some cases. Further large-scale prospective studies will be necessary to confirm the utility of TSS.

## Data Availability

The original contributions presented in the study are included in the article/Supplementary Materials, further inquiries can be directed to the corresponding author/s.
